# Dietary lysine restriction induces lipid accumulation in skeletal muscle through an increase in serum threonine levels in rats

**DOI:** 10.1016/j.jbc.2021.101179

**Published:** 2021-09-08

**Authors:** Yuki Goda, Daisuke Yamanaka, Hiroki Nishi, Masato Masuda, Hiroyasu Kamei, Mikako Kumano, Koichi Ito, Masaya Katsumata, Keitaro Yamanouchi, Naoyuki Kataoka, Fumihiko Hakuno, Shin-Ichiro Takahashi

**Affiliations:** 1 Departments of Animal Sciences and Applied Biological Chemistry, Graduate School of Agricultural and Life Sciences, The University of Tokyo, Tokyo, Japan; 2 Department of Veterinary Medical Sciences, Graduate School of Agricultural and Life Sciences, The University of Tokyo, Tokyo, Japan; 3 School of Veterinary Science, Azabu University, Sagamihara, Kanagawa, Japan

**Keywords:** amino acid, fatty acid, lipid transport, lipid metabolism, muscle, lysine, threonine, fiber twitch, lipid accumulation, amino acid restriction, CN, control, CT, computed tomography, DMEM, Dulbecco's modified Eagle's medium, FATP, fatty acid transport protein, FBS, fetal bovine serum, IMCL, intramyocellular lipid, IMF, intermuscular fat, low-AA, low amino acid, MLT, musculus longissimus thoracis, PBS, phosphate-buffered saline, SOM, self-organizing map, TAG, triglyceride, VLDL, very low-density lipoprotein

## Abstract

We previously reported that dietary amino acid restriction induces the accumulation of triglycerides (TAG) in the liver of growing rats. However, differences in TAG accumulation in individual cell types or other tissues were not examined. In this study, we show that TAG also accumulates in the muscle and adipose tissues of rats fed a low amino acid (low-AA) diet. In addition, dietary lysine restriction (low-Lys) induces lipid accumulation in muscle and adipose tissues. In adjusting the nitrogen content to that of the control diet, we found that glutamic acid supplementation to the low-AA diet blocked lipid accumulation, but supplementation with the low-Lys diet did not, suggesting that a shortage of nitrogen caused lipids to accumulate in the skeletal muscle in the rats fed a low-AA diet. Serum amino acid measurement revealed that, in rats fed a low-Lys diet, serum lysine levels were decreased, while serum threonine levels were significantly increased compared with the control rats. When the threonine content was restricted in the low-Lys diet, TAG accumulation induced by the low-Lys diet was completely abolished in skeletal muscle. Moreover, in L6 myotubes cultured in medium containing high threonine and low lysine, fatty acid uptake was enhanced compared with that in cells cultured in control medium. These findings suggest that the increased serum threonine in rats fed a low-Lys diet resulted in lipid incorporation into skeletal muscle, leading to the formation of fatty muscle tissue. Collectively, we propose conceptual hypothesis that “amino-acid signal” based on lysine and threonine regulates lipid metabolism.

For many years, amino acids have been thought of as the building blocks of protein and a source of energy. Recently, however, amino acids have also been recognized as signal transducers that activate signal transduction pathways (1, 2). Despite intensive research, the mechanisms of amino acid signaling are only just beginning to be elucidated.

It is well established that when growing rats are fed a diet in which the protein content is insufficient, or an essential amino acid is lacking, catabolism is enhanced, whereas anabolism is inhibited, resulting in growth retardation. Concurrently, we previously showed that neutral lipids accumulate in the liver of growing rats fed a diet deficient in protein or arginine (3, 4, 5, 6). In addition, a lysine-deficient diet increases the lipid content in the skeletal muscle of pigs (7). These studies highlight the possibility that amino acids function in the control of lipid metabolism.

In many animals, triglycerides (TAGs) serve as efficient energy storage molecules. Under energy-rich conditions, dietary carbohydrates are converted into fatty acids and stored as neutral lipids (TAG), mainly in adipose tissue, or ectopically in other tissues, including the liver and muscle (8, 9). In contrast, under conditions of starvation, very low-density lipoprotein (VLDL) is released from the liver and circulated in the blood (10). Once in circulation, TAG in VLDL is hydrolyzed by lipoprotein lipase and divided into fatty acids and glycerol (11). Skeletal muscle takes up hydrolyzed fatty acids using fatty acid transporters, including fatty acid transport protein (FATP) 1, FATP4, and CD36 (12). Fatty acids incorporated into skeletal muscles are metabolized and consumed as energy (13).

Intramyocellular lipid (IMCL) is an ectopic fat deposit stored in skeletal muscle cells (14). IMCL is thought to be associated with several disorders, such as type 2 diabetes mellitus, sarcopenia, aging, and obesity (15). However, the underlying mechanism of IMCL remains unknown.

Skeletal muscle is divided into fast-twitch fibers and slow-twitch fibers, and these fibers are also classified by isoforms of myosin heavy chain (MyHC): MyHC I, IIa, and IIb. Myofibers that express MyHC I are slow-twitch fibers, and fibers expressing MyHC IIa/b are fast-twitch fibers (16). It has been reported that MyHC I-expressing slow-twitch fibers are associated with lipid accumulation in skeletal muscle cells (17).

To date, the mechanism by which dietary amino acid deficiency regulates lipid metabolism in the skeletal muscle remains unclear. The skeletal muscle is the largest organ that is a major site of energy production. Accordingly, it is important to understand how skeletal muscles respond to amino acids in a state of malnutrition. In this study, we demonstrated that a low amino acid diet and a low lysine diet induced lipid accumulation not only in adipose tissue but also in the skeletal muscle of growing rats. We also showed that the underlying mechanisms were specific to each diet. In particular, a low lysine diet induced lipid accumulation in skeletal muscle was dependent on changes in the serum amino acid profile that controlled fatty acid transport into muscle cells.

## Results

### Dietary amino acid restriction or lysine restriction induced lipid accumulation in skeletal muscle

**Figure 1:**
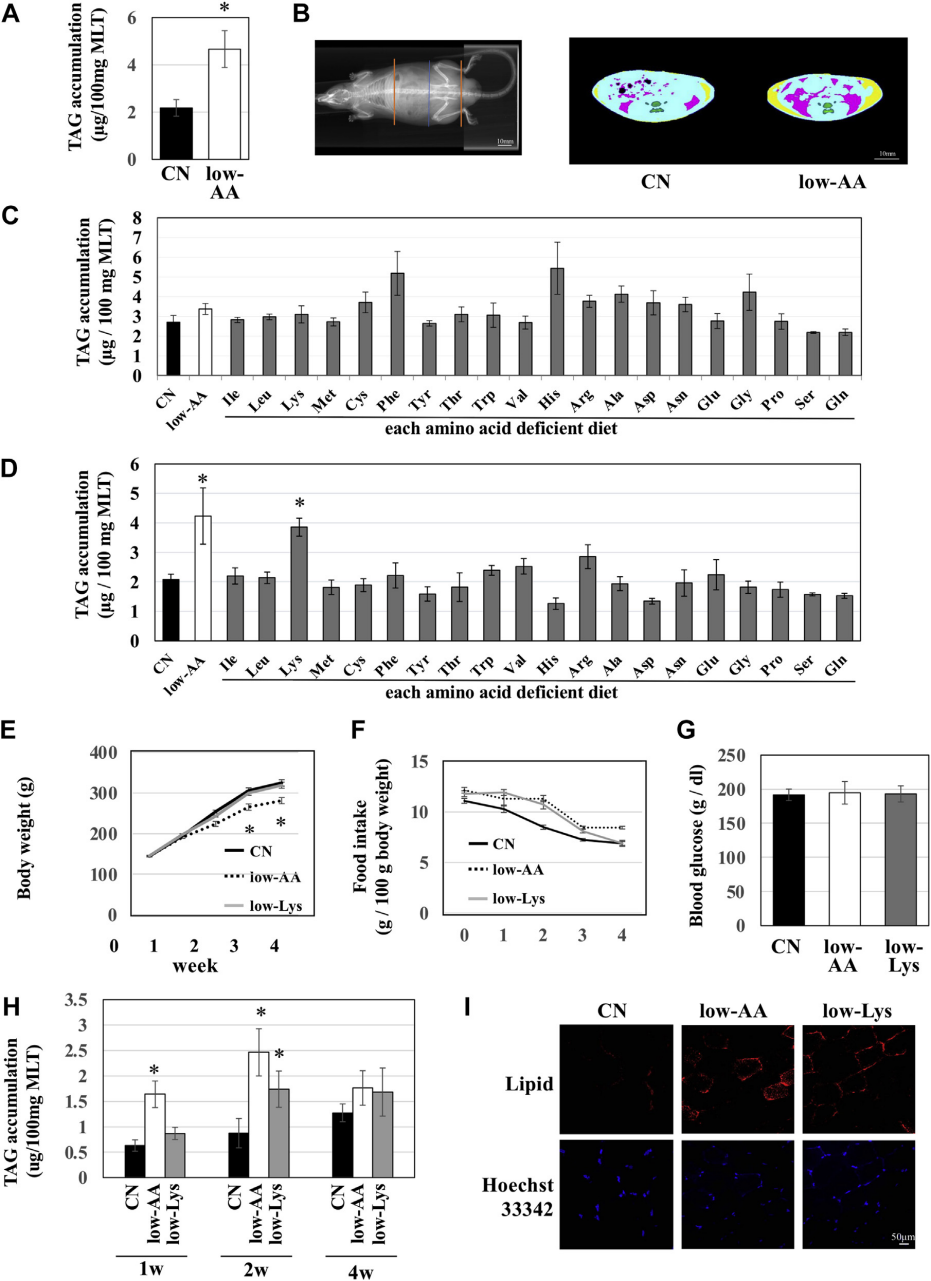
**Lipid accumulation in skeletal muscle of rats fed a low-AA or low-Lys diet.** Six-week-old male Wistar rats were fed experimental diets *ad libitum* for 1 week (*C*), 2 weeks (*A*, *B*, and *D–I*), or 4 weeks (*E*, *F*, and *H*). *A*, *C*, *D*, and *H*, lipids were extracted from the skeletal muscles with methanol-chloroform solution according to Folch's method, and the TAG content was measured. The results are shown as μg TAG per 100 mg wet weight of MLT. n = 3, means ± S.E., ∗*p* < 0.01 *versus* CN. *B*, adipose tissue was measured using an X-ray CT scan. The ends of the scanned region are indicated by *red lines*, and the position of each representative CT scan image is indicated by the *blue line* (*left panel*). In the CT image, subcutaneous fat is shown in *yellow*, and visceral fat is shown in *red* (*right panel*). *E* and *F*, body weight and food intake were measured at 10 AM every day, and the values per 1 week are shown in the graph. n = 4, mean ± S.E. *G*, the blood glucose levels were measured and are shown in the graph. Lipids were stained with Lipid Tox and nucleus with Hoechst33342 in the frozen sections of MLT. Images were obtained using a confocal microscope.

We prepared a control (CN) diet containing a sufficient amount of amino acid powder, equivalent to 15% casein protein, and a low amino acid (low-AA) diet, in which amino acids were reduced to one-third of the CN diet. We fed these diets to 6-week-old rats for 2 weeks and analyzed subsequent lipid accumulation in skeletal muscle. As a result, the TAG content in skeletal muscle tissue (musculus longissimus thoracis, MLT) was higher in rats fed a low-AA diet than in those fed a CN diet (Fig. 1*A*). To investigate the effects of amino acid restriction on adipose tissue, we performed computed tomography (CT) and obtained sectional images of the abdomen in these animals (Fig. 1*B*). The CT scan analysis showed that the areas of both subcutaneous and visceral adipose tissues were increased by feeding the low-AA diet. These data suggest that dietary amino acid restriction induces lipid accumulation in skeletal muscle and adipose tissue.

To determine which amino acid deficiency induces lipid accumulation in the skeletal muscle in response to amino acid restriction, we prepared 20 single amino acid–deficient diets (only one of the 20 amino acids was reduced to the same level as the low-AA diet) and fed them to rats for 1 or 2 weeks. When rats were fed each diet for 1 week, lipid did not accumulate in MLT (Fig. 1*C*). Interestingly, when rats were fed each diet for 2 weeks, TAG accumulation in MLT was induced only in the lysine-restricted diet (low-Lys)-fed group (Fig. 1*D*). The other single amino acid–restricted diet did not enhance lipid accumulation.

Next, rats were fed either the CN diet, or low-AA diet, or low-Lys diet for 1–4 weeks, and the indicated parameters were measured. As shown in Figure 1*E*, rats fed the low-AA diet showed growth retardation, while the body weight of the low-Lys group was similar to that of the CN group (Fig. 1*E*). The food intake per body weight was almost comparable among all groups except at a time point of 2 weeks, at which the food intake was higher in the low-AA and low-Lys groups (Fig. 1*F*). Blood glucose levels were not significantly different between the groups (Fig. 1*G*). The TAG levels in the MLT of the low-AA group were found to increase after 1 week of feeding and were maintained for 4 weeks. In contrast, the TAG content in the MLT of the low-Lys group began to increase by 2 week of feeding and showed a gradual increase until 4 weeks (Fig. 1*H*). Additionally, after staining the neutral lipids in the MLT of the 2-week feeding group using a lipid-binding fluorescent dye, the fluorescent intensities of lipid staining in the low-AA and low-Lys groups were found to be markedly higher than those of the CN group (Fig. 1*I*).

### A lysine-deficient diet induced lipid accumulation in adipose tissue

**Figure 2:**
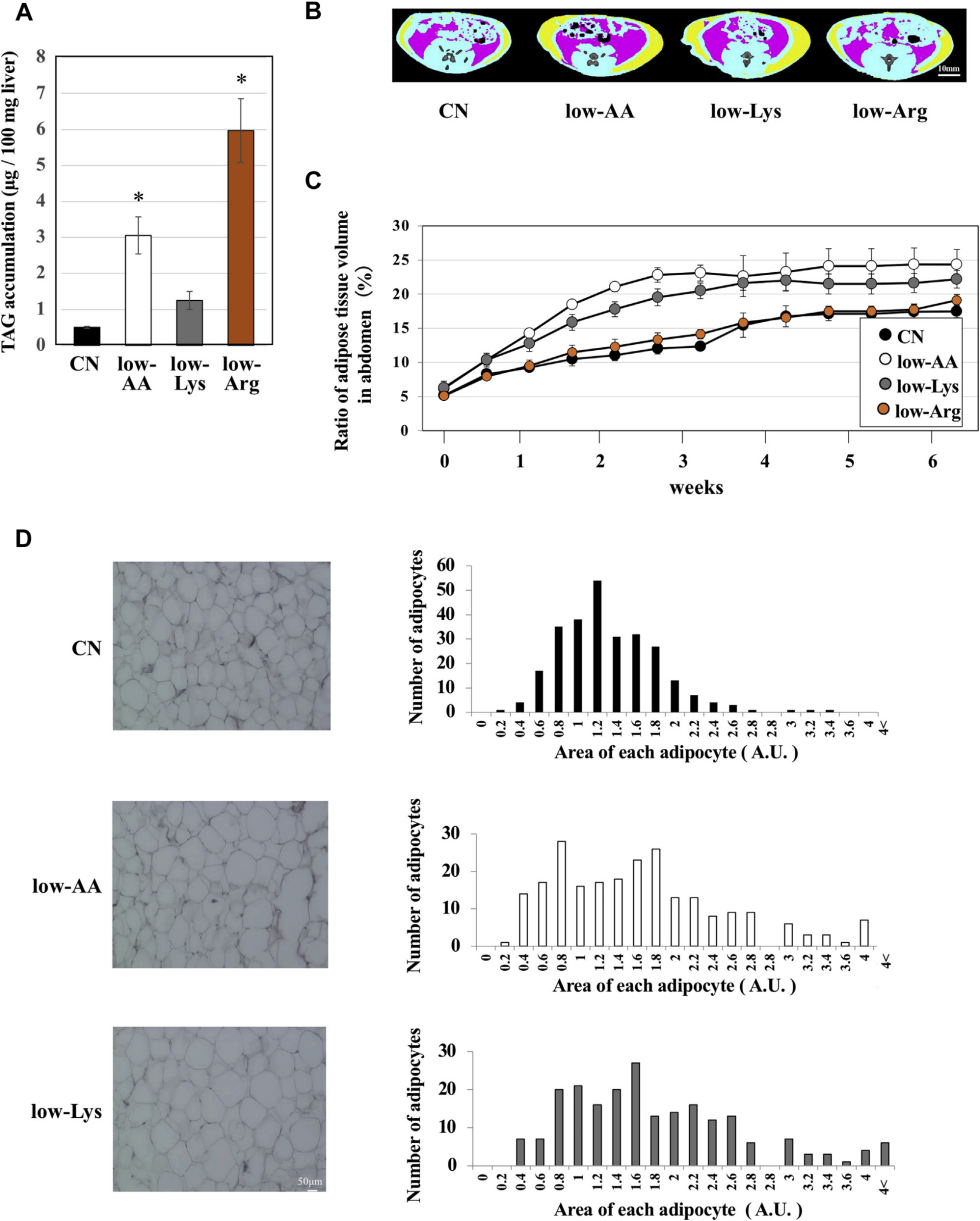
**Low-AA and low-Lys diets increase lipid contents in adipose tissue.** Six-week-old male Wistar rats were fed CN, low-AA, low-Lys, or low-Arg diets *ad libitum* for 1 week (*A*) or 6 weeks (*B*, *C*, and *D*). *A*, lipids were extracted from the livers with methanol-chloroform solution according to Folch's method, and the TAG content was measured. The results are shown as μg TAG per 100 mg wet weight of the liver. n = 4, means ± S.E., ∗*p* < 0.01 *versus* CN. *B* and *C*, sectional images of the abdomen were obtained periodically using an X-ray CT scan. The scanned region was the same as that shown in Figure 1 (indicated by the *red lines* in Fig. 1*B*). Representative CT images are shown. Subcutaneous fat is displayed in *yellow*, and visceral fat is shown in *red*. Areas of the adipose tissue (subcutaneous fat and visceral fat) were integrated using sequential CT scan images of the abdomen, and the percentage of total volume of adipose tissue was calculated and is shown in the graph. n = 3, means ± S.E., ∗*p* < 0.01. *D*, epididymis fat tissues were collected, and paraffin sections were stained with hematoxylin and eosin. The cross-sectional areas of the adipocytes were measured and are shown in the histogram.

We previously reported that lipids accumulate in the liver of rats fed a low-AA or low-Arg diet (4). To investigate this, in the present study, the lipid content in the liver was measured in rats fed each diet. Lipid accumulation in the liver was not observed in rats fed the low-Lys diet for 2 weeks, although the lipid content was found to increase in the low-AA or low-Arg groups (Fig. 2*A*), as previously reported (4). To further investigate the effects of amino acid restriction on adipose tissue, we prepared CN, low-AA, and low-Lys diets, as well as a low-Arg diet. We performed a CT scan and obtained sectional images of the abdomen of rats periodically during the feeding period up to 6 weeks. We calculated the ratio of the adipose tissue volume to the total volume of the abdomen and plotted the values as a graph (Fig. 2, *B* and *C*). These experiments revealed that dietary amino acid or lysine restriction increased the ratio of the adipose tissue volume, while dietary arginine restriction did not.

To evaluate how dietary lysine restriction increased the volume of adipose tissue, we collected epididymal fat tissue from 6-week feeding rats and prepared paraffin sections, followed by hematoxylin and eosin staining to observe morphological changes in adipocytes. The area of each adipocyte in the low-AA and low-Lys groups was larger than that in the CN group, indicating that lysine restriction induced hypertrophy of adipocytes (Fig. 2*D*).

### A low-Lys diet induced lipid accumulation in skeletal muscle in a distinct manner from a low-AA diet

**Figure 3:**
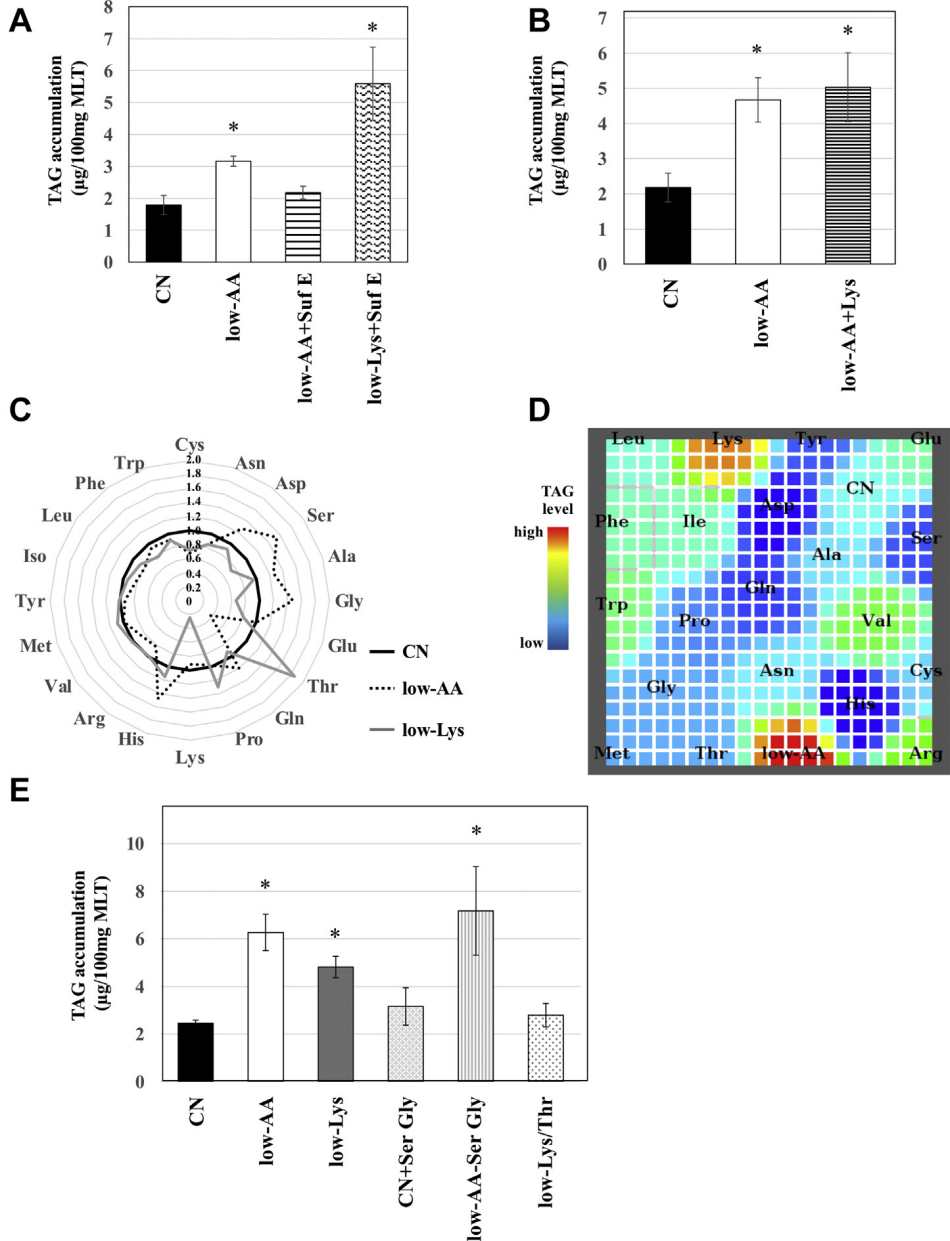
**An increase in serum threonine is required for muscular lipid accumulation in rats fed a low-Lys diet.** Six-week-old male Wistar rats were fed *ad libitum* for 2 weeks with experimental diets, as indicated in *A–C*, *D*, and *E*, or with single amino acid–deficient diets as indicated in Figure 1. *A*, *B*, and *E*, lipids were extracted from the skeletal muscles with methanol-chloroform solution according to Folch's method, and the TAG content was measured. The results are shown as μg TAG per 100 mg wet weight of MLT. n = 3, means ± S.E., ∗*p* < 0.05 *versus* CN. *C*, serum amino acid concentrations were measured using LC MS/MS and are shown in a radar chart as fold of CN. *D*, the serum amino acid profiles were classified by SOM analysis and displayed on a map. Muscular TAG levels are shown in the map as a color index. *Red* indicates high and *blue* indicates low.

Amino acids can be used as nitrogen sources. Therefore, nitrogen deficiency may have caused muscular lipid accumulation in the low-AA group. To address this hypothesis, we prepared two diets, low-AA+SufE and low-Lys+SufE. In these diets, the nitrogen content was adjusted to a level equal to that of the CN diet by adding glutamic acid to the low-AA or low-Lys diet, respectively. TAG accumulation in MLT was almost blocked in rats fed the low-AA+SufE diet, whereas lipid was still accumulated in the MLT of the low-Lys+SufE group (Fig. 3*A*).

To investigate whether muscular lipid accumulation in the low-AA group can be explained only by restriction of dietary lysine, we prepared a low-AA+Lys diet, which is a low-AA diet supplemented with lysine, to ensure the dietary lysine level was equivalent to CN. Lipid content analysis in MLT revealed that the TAG level in the low-AA+Lys group was as high as that in the low-AA group (Fig. 3*B*), indicating that muscular lipid accumulation in the low-AA group was not caused by deficiency of lysine alone. To explore the potential mechanism underlying lipid accumulation in the low-AA and low-Lys groups, we measured the concentrations of amino acids in the serum. The results indicated that the serum lysine concentration was lower in the low-Lys diet group but almost comparable in the low-AA diet group compared with the CN group (Fig. 3*C*). We previously reported that hepatic TAG levels could be classified by serum amino acids in rats fed diets composed of a variety of amino acids using an unsupervised machine learning program, self-organizing map (SOM) (4). Therefore, we performed SOM analysis to examine the relationship between the muscular lipid content and serum amino acid profiles. We used only serum amino acids to create a map for SOM analysis, and muscular TAG levels were plotted on the map as a color index (Fig. 3*D*). SOM analysis showed that low-AA rats and low-Lys rats were classified into two different sites on the map and formed two “hot spots” of high muscular TAG (indicated in red). These results suggest that lipid accumulation in MLT was induced by the low Lys diet through a mechanism distinct from that of the low-AA diet.

As shown in Figure 3*C*, the serum serine and glycine concentrations were higher in the low-AA group than in the CN group. Rats were fed a CN+SerGly diet, in which the amounts of serine and glycine were 3-fold those of CN, and a low-AA-SerGly diet, in which serine and glycine were completely removed from the low-AA diet. Lipid content analysis in MLT demonstrated that neither the addition nor removal of serine and glycine in these diets affected lipid accumulation (Fig. 3*E*).

When focusing on serum amino acid profiles in the low-Lys group, we found that the amount of lysine in the serum was significantly decreased, while that of threonine was significantly increased compared with that in the CN group (Fig. 3*C*). To determine whether the increase in serum threonine was required for lipid accumulation in MLT, rats were fed a low-Lys/Thr diet in which both lysine and threonine were reduced to the same level as the low-AA diet. The low Lys diet induced lipid accumulation in MLT, and interestingly, the low-Lys/Thr diet did not show this effect (Fig. 3*E*).

### Dietary lysine restriction increased oxidative muscle fibers, and an amino acid profile mimicking the low-Lys serum increased fatty acid uptake

**Figure 4:**
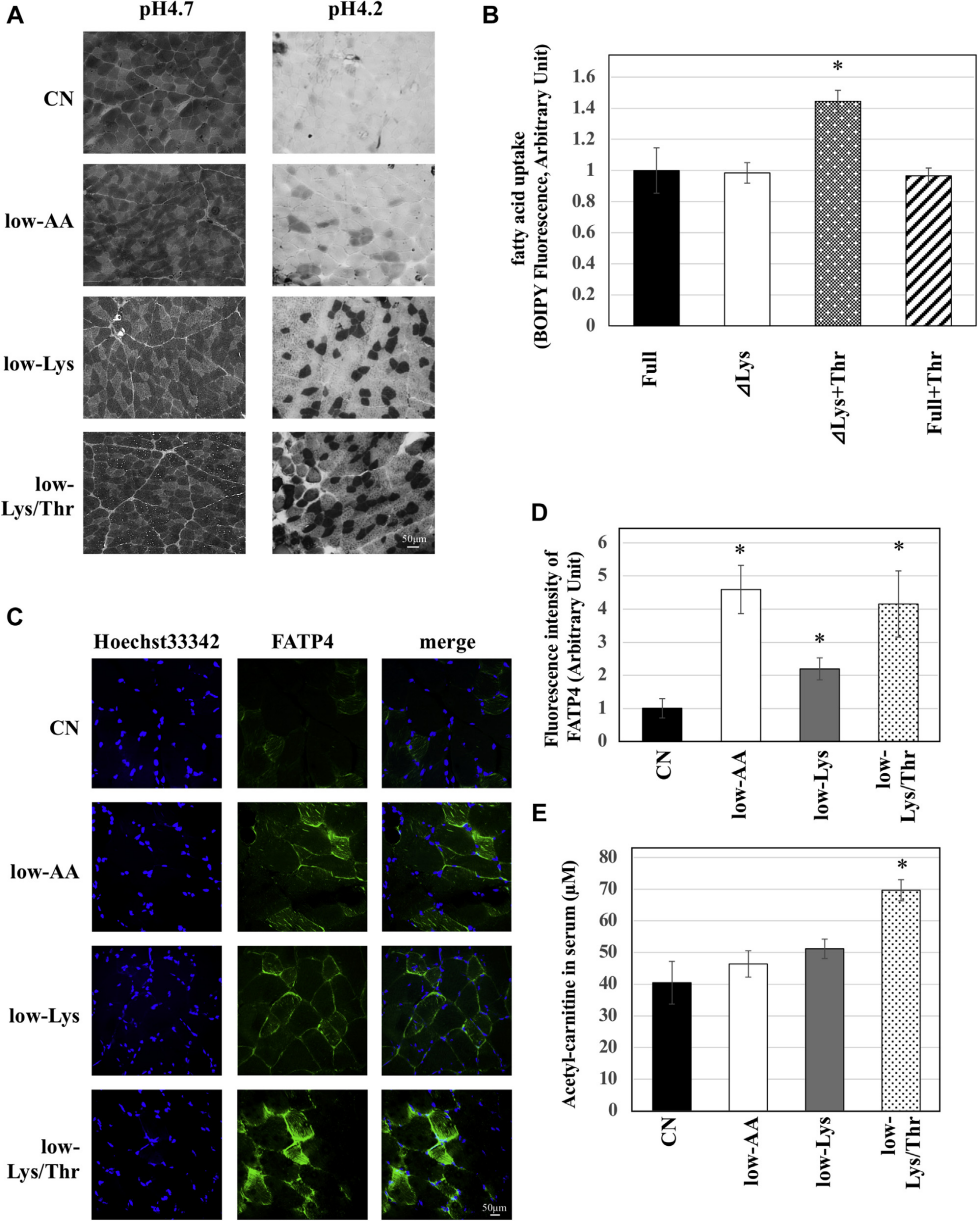
**A low-Lys diet increases oxidative muscle fibers, and an amino acid profile mimicking the low-Lys serum enhances fatty acid uptake.***A*, six-week-old male Wistar rats were fed experimental diets *ad libitum* for 2 weeks. Frozen muscle sections were stained with ATPase staining preincubated with sodium barbital solution adjusted to pH 4.7 or 4.2 for 5 min to stain slow-twitch or fast-twitch. At pH 4.2, myosin, except for the type I myosin ATPase, is inactivated. Thus, type I myosin fibers are strongly stained as *black* at pH 4.2. *B*, well-differentiated L6 cells were cultured in the experimental medium for 2 days, and 100 μM BODIPYFLC_12_ were added to the cells during the last 4 h. Lipids were extracted by Folch's method and fluorescent intensity was measured (n = 3, means, ±S.E.) ∗*p* < 0.01, *versus* Full medium. *C* and *D*, frozen sections of MLT were immuno-stained with anti-FATP4 antibody (*green*), and nucleus was stained with Hoechst33342 (*blue*). The images were obtained with the confocal microscope (*C*). The green fluorescent intensity of each picture was quantified by Image J. Values were shown in the graph (*D*). n = 3, means ± S.E., ∗*p* < 0.01 *versus* CN. *E*, acetyl-carnitine concentration in the serum from each rat was measured using LC MS/MS (n = 3, means ± S.E.) ∗*p* < 0.01 *versus* CN.

Several studies have found that the muscular lipid content varies depending on the type of myofiber in pigs, mice, and humans (17, 18, 19). Thus, next, we assessed the possibility that the difference in myofiber type is involved in lipid accumulation in skeletal muscle. We performed myosin ATPase staining in the MLT of rats fed the CN, low-AA, low-Lys, or low-Lys/Thr diet. MLT sections were pretreated with pH 4.2 or 4.7 buffers, and different types of myosin ATPase were inactivated according to pH. Under pH 4.2, myosin, except for type I myosin ATPase, was inactivated; thus, type I myosin fibers were strongly stained black. As shown in Figure 4*A*, the number of type I myosin fibers (strongly stained black) was increased in the low-AA group (Fig. 4*A*). An increase in type I myosin fibers was also observed in the low-Lys group, and this increase was not inhibited by threonine restriction in combination with lysine restriction (low-Lys/Thr) (Fig. 4*A*).

**Figure 5:**
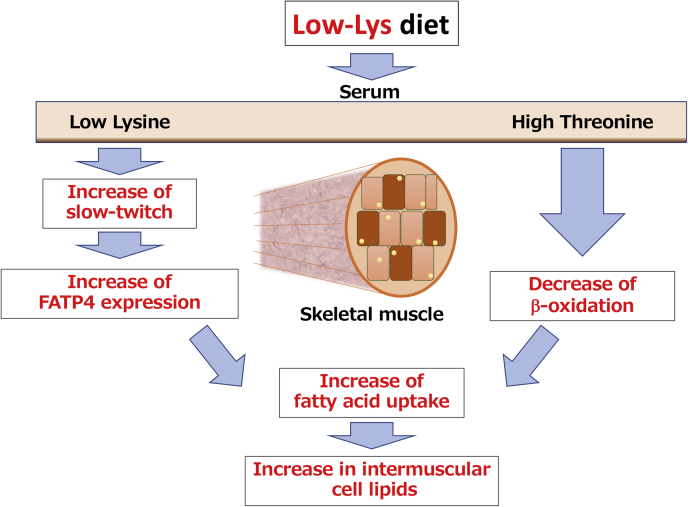
**Working hypothesis of a novel regulatory mechanism of the muscular lipid accumulation in rats fed a low-Lys diet.** In the rats fed a low-Lys diet, the serum lysine levels are low while the serum threonine levels are high. Low levels of serum lysine may increase the ratio of slow-twitch muscle fibers, leading to increase in FATP4 expression. On the other hand, high serum threonine levels may inhibit β-oxidation *via* an unknown mechanism. These two changes in serum amino acid level are required for the increase in the fatty acid uptake, leading to intermuscular cell lipids.

Finally, fatty acid uptake in myotubes was examined using cell lines. Differentiated L6 myotubes were cultured in medium containing four types of amino acid profile: full medium, which contains sufficient amounts of amino acids, ΔLys medium containing sufficient amino acids except that lysine is depleted, ΔLys+Thr medium, which is a ΔLys medium containing 3-fold threonine, and Full+Thr, which is a full medium containing 3-fold threonine. BODIPY FL C12, a fatty-acid-conjugated fluorescent dye, was added to the medium during the final 4 h to measure fatty acid uptake into the cells. Fatty acid uptake was unchanged in cells cultured in ΔLys or Full+Thr medium compared with cells cultured in the full medium (Fig. 4*B*). Interestingly, lysine deficiency in combination with excess threonine (ΔLys+Thr), which mimics the serum amino acid profile in low-Lys rats, increased fatty acid uptake compared with control cells (Fig. 4*B*). These data indicate that fatty acid uptake into muscle cells was enhanced by culturing in ΔLys+Thr medium. It is well known that muscular lipid accumulation is caused by lipid uptake from the blood. Thus, we examined the expression levels of fatty acid transporters in MLT. We performed immunostaining analysis of the fatty acid transporter FATP4 in MLT and measured the quantity of fluorescence (Fig. 4, *C* and *D*). The FATP4 expression levels were increased in the low-AA, low-Lys, and low-Lys/Thr groups. Although these data suggest that the low-AA, low-Lys, and low-Lys/Thr diets increased fatty acid transporters, this is in conflict with the data that the low-Lys/Thr diet did not accumulate TAG in skeletal muscle (Fig. 3*E*). Acetyl-carnitine in serum, which is a biomarker of β-oxidation in skeletal muscle, was increased by the low-Lys/Thr diet (Fig. 4*E*), suggesting that β-oxidation was activated in rats fed the low-Lys/Thr diet. These data suggest that both FATP4 expression and β-oxidation were enhanced in rats fed the low-Lys/Thr diet, resulting in the inhibition of lipid accumulation (Fig. 5).

## Discussion

In this study, we demonstrated that a low-AA diet and a low-Lys diet increased the neutral lipid content in skeletal muscle and adipose tissues. We further focused on muscular lipid accumulation to elucidate the mechanisms underlying metabolic changes in skeletal muscle. We found that oxidative slow-twitch muscle fibers were associated with dietary amino acid or lysine deficiency, which may contribute to the increased lipid content. Importantly, dietary lysine deficiency increased serum threonine levels, which are required for lipid accumulation in the skeletal muscle. We hypothesized that: (1) dietary lysine deficiency increased slow-twitch muscle fibers, which might be involved in the high expression of FATP4; (2) concomitantly, dietary lysine deficiency not only decreased serum lysine but also increased serum threonine, and this low lysine/high threonine status is important for cell-autonomous induction of fatty acid uptake in muscle cells; (3) these two pathways could coordinately function to positively control lipid metabolism in skeletal muscle (Fig. 5).

Rats were fed with 20 types of single amino acid–deficient diets for 2 weeks in order to identify the amino acid responsible for muscular lipid accumulation. This screening experiment provided us with data showing that lysine deficiency alone induced lipid accumulation in MLT (Fig. 1*D*). It is worth noting that the lysine-deficient diet required 2 weeks to increase muscular lipid content, while this diet did not induce lipid accumulation in MLT at a 1-week time point (Fig. 1*C*). These results suggest that L-Lys-dependent lipid accumulation requires a long-term feeding period.

In addition, we also found that the low-AA diet and the low-Lys diet increased the total volume of adipose tissue in the abdomen (Fig. 2). This increase in adipose tissue is associated with the enlargement (hypertrophy) of adipocytes. Meanwhile, the low-Arg diet induced lipid accumulation in the liver, but not in the muscle or adipose tissue (Fig. 2). These results indicate that the increase in the lipid content in these tissues is specific to lysine-deficient states. Thus, we speculate that dietary lysine deficiency positively controls cell differentiation and/or lipid metabolism in adipocytes. It is well known that adipocytes exist in the interspace between muscle fibers (intermuscular fat, IMF) (20, 21). Thus, these adipocytes may have contributed in part to the increased lipid accumulation in skeletal muscle observed in rats fed a low-AA or low-Lys diet.

The low-AA and low-Lys diets contained the same amount of lysine. Thus, we initially expected that the muscular lipid accumulation observed in both low-AA and low-Lys groups could be explained by a shortage of dietary lysine content. Contrary to our expectation, feeding rats with a low-AA+Lys diet, which is a low-AA diet supplemented with a sufficient amount of lysine, induced lipid accumulation in skeletal muscle (Fig. 3*B*). These results suggest that the low-AA and low-Lys diets induced lipid accumulation through different mechanisms. Indeed, the supplementation of glutamic acid as a nitrogen source to the low-AA diet and the low-Lys diet also had different effects on lipid accumulation (Fig. 3*A*). In order to determine the mechanistic differences between the low-AA and low-Lys diet groups, we examined the amino acid profiles in the serum and found that: (1) the low-AA diet increased serum serine and glycine; (2) the low Lys diet not only reduced lysine but also increased threonine in serum (Fig. 3*C*). We did not obtain a clear result indicating that the changes in serine and glycine in the low-AA group were responsible for muscular lipid accumulation. However, our results that a dietary restriction in both lysine and threonine did not induce lipid accumulation indicate that the increase in threonine in serum was necessary for the low-Lys-induced lipid accumulation.

Our analysis of a type of myosin ATPase, which is generally believed to be associated with muscular lipid content, revealed that the low Lys diet increased type I myosin fibers (slow-twitch myofibers). It has been reported that slow-twitch myofibers contain more lipids than fast-twitch myofibers (17). This may be one of the reasons for lipid accumulation in skeletal muscles. In this study, we used MLT, which is generally known as a fast-twitch rich myofiber. MLT has a greater capacity for fiber type transformation, resulting in greater lipid accumulation. Our results show that the low-Lys diet increased slow-twitch myofibers irrespective of the dietary threonine content (Fig. 4*A*), suggesting that an increase in slow-twitch myofibers is one of the necessary conditions for lipid accumulation, although not a sufficient condition. Consistently, our cell culture experiments showed that fatty acid transport was enhanced in L6 myotubes only under a lysine-deficient and threonine-excess condition (which mimics a serum amino acid profile in the low-Lys group) (Fig. 4*B*). These results suggest that the low Lys diet upregulated lipid accumulation in skeletal muscle by increasing fatty acid transport through a cell-autonomous mechanism. FATP4, a typical fatty acid transporter in skeletal muscle, was increased in the MLT of low-AA, low-Lys, and low-Lys/Thr diets. FATP4 is known to be increased mainly in slow-twitch muscle (22), such that an increase in slow-twitch fibers caused by deficiency of lysine may increase FATP4. However, fatty acid intake was increased only after harvesting L6 cells in low- and high-Thr medium (Fig. 4*B*). Serum acetyl-carnitine is related to β-oxidation in the skeletal muscle (23). Therefore, the increase in serum acetyl-carnitine (Fig. 4*D*) suggests that the β-oxidation of skeletal muscle in the low-Lys/Thr diet group was much more activated than in the low-AA and low-Lys diet groups. These data suggest that dietary lysine restriction increased fatty acid intake and induced lipid accumulation, but that the deficiency of both lysine and threonine activated β-oxidation through an unknown mechanism. Thus, the dietary restriction of both lysine and threonine increases the consumption of lipids, resulting in the inhibition of lipid accumulation in skeletal muscle.

Insulin stimulates glucose uptake and induces lipid accumulation in healthy individuals. The IMCL is used as an energy source during exercise (24). However, in obese individuals with type 2 diabetes, excessive IMCL is negatively correlated with compromised plasma fatty acids, which induces insulin resistance in skeletal muscle (25, 26). Paradoxically, in endurance-trained athletes, insulin sensitivity is very high in skeletal muscles, despite high levels of IMCL (27). This phenomenon is known as the athlete's paradox. In our study, a low-AA diet and a low-Lys diet increased IMCL in skeletal muscle but did not change the blood glucose levels (Fig. 1*G*). Additionally, these diets increased oxidative slow-twitch fibers (Fig. 4*A*), which have high energy activity. Taken together, the low-AA or the low-Lys diet induced insulin-sensitive IMCL, similar to endurance-trained athletes.

We demonstrated that specific amino acid profiles induce lipid accumulation in skeletal muscle and adipose tissue. Our results provide us with the possibility of developing a novel technique to control lipid-accumulating tissues and accumulate lipids by changing serum amino acid profiles. The development of this technique could lead to applied studies, including livestock science studies that modify the quality of meat by increasing or decreasing lipid content, or medical science studies that prevent or treat metabolic syndrome. We expect that our study will be the basis for these applied studies.

## Experimental procedures

### Materials

For the experimental diets, a vitamin mixture, cellulose powder, and corn starch were purchased from Oriental Yeast, and soybean oil was purchased from Nacalai Tesque. For cell culture medium, Earle's buffered salt solution (EBSS) and MEM vitamin solution were purchased from Sigma Aldrich. Dulbecco's modified Eagle's medium (DMEM) and phosphate-buffered saline (PBS) were purchased from Nissui Pharmaceutical Co. Fetal bovine serum (FBS) was purchased from Sigma–Aldrich. Penicillin and streptomycin were purchased from Banyu Pharmaceutical Co.

### Animals

Wistar rats (5-week-old, male) were purchased from Charles River Japan. These rats were caged individually and kept in a room maintained at 24 ± 1 °C with 50–60% humidity and a 12 h light (8:00–20:00)/dark (20:00–8:00) cycle. We prepared a control experimental diet (CN) and each experimental diet. The CN diet was composed of 15% amino acids (detailed composition is shown in Table 1). In each experimental diet, the total amino acids or amino acids were restricted to 1/3 (w/w) of the CN diet or increased. These rats were prefed normal chow for 3 days and a CN diet for 4 days before carrying out our experiment as a training. After prefeeding, we started feeding the experimental diets for the weeks required for each experiment. During the experimental period, the body weight and food intake of all rats were measured daily at 10:00 AM. For tissue collection, rats were anesthetized with isoflurane (DS Pharma Animal Health) after 1 h of fasting. Then, blood from the carotid artery and skeletal muscle, adipose tissue, and liver of each rat were collected. MLT was selected as the representative skeletal muscle in this study. All animal care and experiments conformed to the Guidelines for Animal Experiments of the University of Tokyo and were approved by the Animal Research Committee of the University of Tokyo.

**Table 1:** Compositions of experimental diets

Component	CN	Low-AA	Low-Lys	Low-Lys/Thr	Low-Arg	CN+SerGly	Low-Ser/Gly
L-Isoleucine	7.1	2.7	7.1	7.1	7.1	7.1	7.1
L-Leucine	13.0	4.3	13.0	13.0	13.0	13.0	13.0
L-Lysine·HCl	14.1	3.7	3.7	3.7	14.1	14.1	3.7
DL-Methionine	6.4	2.1	6.4	6.4	6.4	6.4	6.4
L-Cystine	0.8	0.3	0.8	0.8	0.8	0.8	0.8
L-Phenylalanine	7.2	2.4	7.2	7.2	7.2	7.2	7.2
L-Tyrosine	7.8	2.6	7.8	7.8	7.8	7.8	7.8
L-Threonine	6.1	3.0	6.1	3.0	6.1	6.1	6.1
L-Tryptophan	1.7	0.6	1.7	1.7	1.7	1.7	1.7
L-Valine	9.2	3.1	9.2	9.2	9.2	9.2	9.2
L-Histidine	4.1	1.4	4.1	4.1	4.1	4.1	4.1
L-Arginine	5.2	1.7	5.2	5.2	1.7	5.2	5.2
L-Alanine	4.1	1.4	4.1	4.1	4.1	4.1	4.1
L-Aspartic acid	5.1	1.7	5.1	5.1	5.1	5.1	5.1
L-Asparagine	5.8	1.9	5.8	5.8	5.8	5.8	5.8
L-Glutamic acid	14.6	4.9	14.6	14.6	14.6	14.6	14.6
Glycine	2.6	0.9	2.6	2.6	2.6	7.8	0
L-Proline	15.0	5.0	15.0	15.0	15.0	15.0	15.0
L-Serine	8.1	2.7	8.1	8.1	8.1	24.3	0
L-Glutamine	14.6	4.9	14.6	14.6	14.6	14.6	14.6
Cellulose	100	100	100	100	100	100	100
Vitamin mixture	10	10	10	10	10	10	10
Mineral mixture	40	40	40	40	40	40	40
Soybean oil	50	50	50	50	50	50	50
Corn starch	647.5	748.7	657.8	660.9	650.9	626	657.8
Total	1000	1000	1000	1000	1000	1000	1000

### Measurement of muscle TAG

We extracted total lipids in skeletal muscle according to Folch's method (28). Briefly, frozen tissue was homogenized with methanol-chloroform solution (1:1, v/v) and left overnight, after which 1/4 volumes of 0.8% KCl were added and centrifuged at 13,000*g* at 4 °C for 10 min. The organic layer was collected, evaporated, and dissolved in isopropanol. The TAG content in the lipid extract was measured using a triglyceride E-test (WAKO).

### Immunostaining and histology

Collected skeletal muscle tissue was divided and frozen in isopentane cooled with liquid nitrogen and dried on dry ice for 2 h. Cryosections (7 μm thickness) were prepared using a cryostat, air dried, and stored at ‒80 °C. When using the tissue sections, these were air dried again. For lipid staining, the tissue sections were fixed in 4% paraformaldehyde, washed in PBS three times, and stained with Lipid Tox Red (Thermo Fisher Scientific). For myosin ATPase staining, the tissue sections were preincubated in 0.1 M sodium barbital solution adjusted to pH 4.7 and 4.2 for 5 min and washed twice in DW. Next, they were incubated in ATP-incubating-solution adjusted to pH 9.45 (60 mg ATP powder, 6 ml 0.1 M sodium barbital, and 3 ml CaCl_2_ in 21 ml of DW) for 25 min, washed three times in 1% CaCl_2_ solution, and incubated in 2% CoCl_2_ solution for 5 min. Then, these were washed in 0.05 M barbital solution and incubated in 2% NH_4_SO_3_ solution for 30 s. For immunostaining, the tissue sections were fixed in 4% paraformaldehyde, incubated in PBS containing 10% octylphenol ethoxylate for 15 min, washed twice in PBS, and treated with 5% goat serum for 60 min at RT. The sections were then incubated with primary antibodies at 4 °C overnight, followed by secondary staining.

### Serum amino acid analysis

Serum samples (50 μl) were mixed with 120 μl of methanol containing 25 μM 2-(N-morpholino)-ethanesulfonic acid (MES) and 100 μM methionine sulfone as an internal control. After centrifugation (16,000*g* for 10 min at 4 °C), 130 μl of the supernatant was mixed with 250 μl of ultrapure water and subjected to ultrafiltration using 3-kDa cutoff filters (Amicon Ultra 3 K device; Merck), followed by 30 min evaporation and 6 h lyophilization. The lyophilized metabolite samples were reconstituted with 200 μl of ultrapure water, further diluted if necessary, and then subjected to LC-MS/MS (LCMS-8030; Shimadzu) analysis, with Method Package for Primary Metabolites ver. 2 (Shimadzu) according to the manufacturer's protocol.

### Self-organizing map (SOM) analysis

A self-organizing map (SOM), an unsupervised machine learning program proposed by Kohonen (29), can classify high-dimensional datasets, putting them in a lower-dimensional space according to their similarity to each other. Here, RPSOM (30), which is an improved SOM, was used. RPSOM can create better maps by properly selecting the optimal SOM learning parameters. On the map, “similar” datasets can be found close to each other. In a previous study, we reported that rats could be smoothly classified by SOM, using individual serum amino acid concentrations and hepatic TAG levels as input vectors (3).

The initial learning parameters were set such that the learning coefficient had three patterns (0.2→0.01, 0.4→0.1, 0.5→0.2) and the neighborhood radius had three patterns (5→1, 10→2, 20→4). The number of training iterations was 1000, and the size of the map was 20 × 20. The optimal learning parameters were selected for each iteration. In addition, the edges of the map were wall boundaries, not periodic boundaries. As such, they did not interfere with the outside.

We carried out SOM analysis using only serum amino acid concentrations as input data without TAG in the musculus longissimus thoracis. Using this analysis, we obtained a 20 × 20 square map on which rats with similar serum amino acid profiles were successfully placed near each other. The TAG levels in the MLT of each rat are shown on the map as a heatmap. At this time, two peaks were observed at the TAG level. The two peaks were separated from each other and were considered to belong to different groups.

### Cell culture

L6 rat myoblasts were grown in DMEM supplemented with 10% FBS and antibiotics under 5% CO_2_ at 37 °C. For differentiation into myotubes, the subconfluent L6 myoblasts were cultured in DMEM supplemented with 2% FBS for at least 4 days. After the cells were sufficiently differentiated into myotubes, the differentiated cells were used for the fatty acid uptake assay.

### Fatty acid uptake assay

Differentiated L6 myotubes were cultured in the indicated experimental media for 2 days, and BODIPY FL C12, a fatty acid-conjugated fluorescent dye, was added during the final 4 h. Then, the fluorescence of BODIPY FL C12 in the cells was measured as fatty acid uptake.

### Statistical analysis

Data are expressed as the mean ± standard error of the mean (SEM). Comparisons between two groups were performed using Student's *t* test. Comparisons among more than two groups were performed using one-way analysis of variance (ANOVA). If the *p*-value obtained from the ANOVA test was *p* < 0.05, the Tukey–Kramer post-hoc test was performed. Statistical significance was set at *p* < 0.05. All statistical calculations were performed using JMP Pro software (SAS Institute Inc).

## Data availability

All data are contained within the manuscript.

## Conflict of interest

The authors declare that they have no conflicts of interest with the contents of this article.
